# Characterization and Evolutionary Implications of the Triad Asp-Xxx-Glu in Group II Phosphopantetheinyl Transferases

**DOI:** 10.1371/journal.pone.0103031

**Published:** 2014-07-18

**Authors:** Yue-Yue Wang, Yu-Dong Li, Jian-Bo Liu, Xin-Xin Ran, Yuan-Yang Guo, Ni-Ni Ren, Xin Chen, Hui Jiang, Yong-Quan Li

**Affiliations:** 1 College of Life Sciences, Zhejiang University, Hangzhou, Zhejiang, China; 2 Department of Bioengineering, Zhejiang Gongshang University, Hangzhou, Zhejiang, China; 3 Key Laboratory of Microbial Biochemistry and Metabolism Engineering of Zhejiang Province, Hangzhou, Zhejiang, China; Universität Stuttgart, Germany

## Abstract

Phosphopantetheinyl transferases (PPTases), which play an essential role in both primary and secondary metabolism, are magnesium binding enzymes. In this study, we characterized the magnesium binding residues of all known group II PPTases by biochemical and evolutionary analysis. Our results suggested that group II PPTases could be classified into two subgroups, two-magnesium-binding-residue-PPTases containing the triad Asp-Xxx-Glu and three-magnesium-binding-residue-PPTases containing the triad Asp-Glu-Glu. Mutations of two three-magnesium-binding-residue-PPTases and one two-magnesium-binding-residue-PPTase indicate that the first and the third residues in the triads are essential to activities; the second residues in the triads are non-essential. Although variations of the second residues in the triad Asp-Xxx-Glu exist throughout the whole phylogenetic tree, the second residues are conserved in animals, plants, algae, and most prokaryotes, respectively. Evolutionary analysis suggests that: the animal group II PPTases may originate from one common ancestor; the plant two-magnesium-binding-residue-PPTases may originate from one common ancestor; the plant three-magnesium-binding-residue-PPTases may derive from horizontal gene transfer from prokaryotes.

## Introduction

Phosphopantetheinyl transferases (PPTases) play an essential role in both primary and secondary metabolism [1]–[3]. Recently, the production of natamycin, an antifungal reagent, has been optimized through engineering of a PPTase in an industrial natamycin producer, *Streptomyces chattanoogensis* L10 [4]. PPTases transfer the phosphopantetheinyl group of coenzyme A (CoA) to a conserved serine residue in acyl carrier proteins (ACPs) in fatty acid synthases (FASs) and polyketide synthases (PKSs) as well as peptidyl carrier proteins (PCPs) in nonribosomal peptide synthetases (NRPSs), converting ACPs/PCPs from inactive apo-forms into active holo-forms [5]–[15]. PPTases can be classified into three groups based on their structures. The group I PPTases (ACPS-type PPTases) are about 120 amino acids in length, which form trimeric quaternary structures [8], [11]–[12], [16]. The group II PPTases (Sfp-type PPTases) are more than 220 amino acids in length, which form monomeric tertiary structures with 2-fold pseudosymmetry within the monomers [5], [13]–[15]. The group III PPTases exist as domains of FASs and PKSs [17]–[18].

The group I PPTases are found in most organisms except animals; the group II PPTases exist in almost all organisms; the group III PPTases are only found as domains fused within FASs in fungi and some PKSs in *Streptomyces* [1]. In most bacteria, group I PPTases phosphopantetheinylate ACPs in FASs and group II PPTases phosphopantetheinylate ACPs/PCPs in PKSs/NRPSs [4], [6], [19]. Animals and few bacteria contain single group II PPTase, which phosphopantetheinylate ACPs from both primary metabolism and secondary metabolism [20]–[22]. The group III PPTases phosphopantetheinylate the ACPs which locate with the group III PPTases in the same peptides [17]–[18].

**Figure 1:**
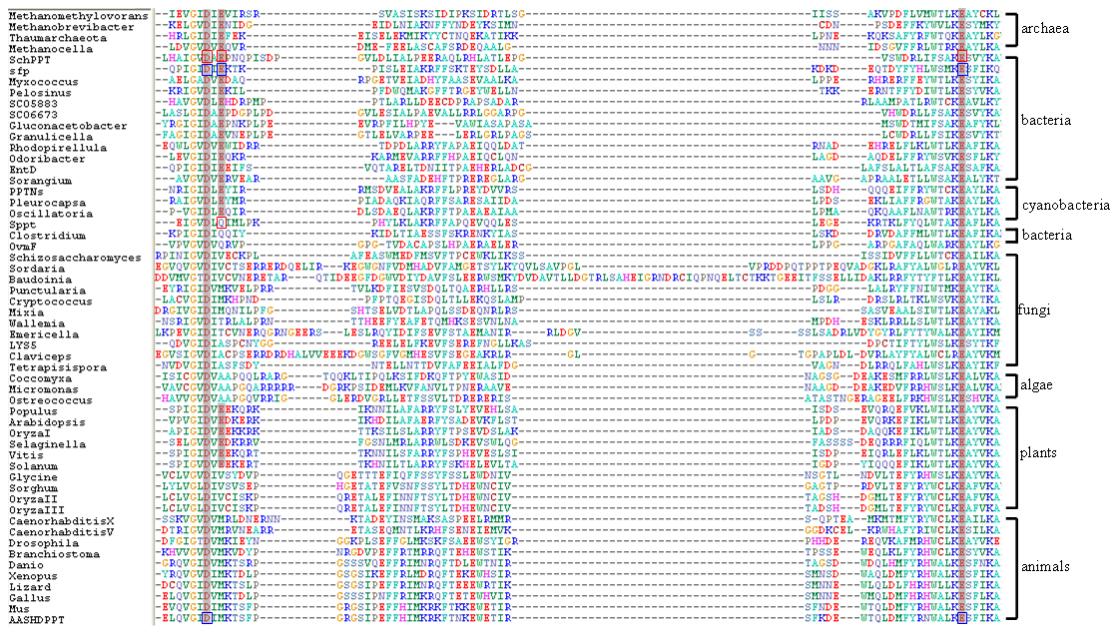
Protein sequence alignment of the 56 group II PPTases selected for phylogenetic analysis. The magnesium binding residues of Sfp and AASHDPPT are in blue frames. The proposed magnesium binding residues are shaded. The mutated residues of SchPPT and Sppt in this study are in red frames.

Magnesium ion is essential to PPTase activity [23]–[24]. X-ray crystal structure analyses of two group II PPTases, Sfp from *Bacillus subtilis* and AASHDPPT from *Homo sapiens*, reveal that one group II PPTase binds one magnesium ion. Six ligands of magnesium ion in Sfp are two phosphates of the CoA, one water molecule, and carboxylates of Asp107, Glu109, and Glu151 of Sfp [24]. Interestingly, although the overall structure of AASHDPPT closely resembles that of Sfp, six ligands of the magnesium ion are two phosphates of the CoA, two water molecules, and carboxylates of Asp129 and Glu181 of AASHDPPT. The two magnesium binding residues of AASHDPPT, Asp129 and Glu181, correspond to the first and the third magnesium binding residues of Sfp (Figure 1) [25]. Sfp and AASHDPPT represent the three-magnesium-binding-residue-PPTases and the two-magnesium-binding-residue-PPTases, respectively.

To understand the relationship between structure and activity and the evolution of PPTases will shed a light on catalytic mechanisms of PPTases. Here, we carried out a systematically evolutionary analysis and a biochemical analysis of group II PPTases. Our results suggested that: (i) group II PPTases could be classified into two subgroups, two-magnesium-binding-residue-PPTases with the triad Asp-Xxx-Glu and three-magnesium-binding-residue-PPTases with the triad Asp-Glu-Glu; (ii) the first and the third residues in the triads are essential to enzyme activities; the second residues in the triads are non-essential; (iii) the animal group II PPTases may originate from one common ancestor; the plant two-magnesium-binding-residue-PPTases may originate from one common ancestor; the plant three-magnesium-binding-residue-PPTases may derive from horizontal gene transfer from prokaryotes.

## Materials and Methods

### Data collection

Protein sequences of annotated PPTases of *E. coli*, *Streptomyces*, and *Homo sapiens* were obtained from the National Center for Biotechnology Information (NCBI) database and were used as queries for gene search using BLASTP, PSI-BLAST from NCBI protein NR databases, with e value 1e-6 as the cutoff. PPTase homologs were selected based on the following criterion: sequence identity >35%, and length coverage >70%. In order to obtain all available annotated PPTases, archaea PPTases, cyanobacterial PPTases, plant PPTases, and animal PPTases were also obtained by searching for the annotated sequences as phosphopantetheinyl transferases from GenBank databases, Phytozome (http://phytozome.net), and Ensembl (http://www.ensembl.org). PPTase data from both methods were merged, and representative sequences was used for further analysis.

### Co-expression of *scn ACP0-2* with *SchPPT* or each of SchPPT point mutant genes

All strains and plasmids used in this study are listed in Table 1. All primers used in this study are listed in Table S1. Plasmid pET44a (Novagen) was digested with *Nde*I/*Hin*dIII, filled in 5′ overhangs to form blunt ends with DNA polymerase Klenow fragment, and then self-ligated with T4 DNA ligase, yielding plasmid pYY0040. The plasmid pYY0040 was introduced into *E. coli* BL21(DE3) containing pHJ0021 [4], in which *scn ACP0-2* was cloned as a *Nde*I/*Hin*dIII fragment into pET28a (Novagen). BL21(DE3)/pHJ0021/pYY0040 was induced with 0.4 mM isopropyl-β-D-thiogalactopyranoside (IPTG) at 30 °C for 4 h to overproduce *scn* ACP0-2. The *scn* ACP0-2 was purified by affinity chromatography on Ni-NTA agarose (Qiagen) and then dialyzed against 20 mM Tris·HCl (pH8.0), 25 mM NaCl, 1 mM dithiothreitol (DTT), and 10% glycerol. The *scn* ACP0-2 was analyzed by LC-MS as described previously [4].

**Table 1:** Plasmids and strains used in this study.

	Descriptions	Reference
Strains		
*S. chattanoogensis* L10	An natamycin producing strain	[4], [28]–[30]
sHJ007	*SchPPT* in-frame deletion mutant of L10	This study
sHJ008	Complementation of *SchPPT* in sHJ007	This study
sHJ009	Complementation of *SchPPT_D105A_* in sHJ007	This study
sHJ010	Complementation of *SchPPT_E151A_* in sHJ007	This study
sHJ011	Complementation of *SchPPT_E107A_* in sHJ007	This study
sHJ012	Complementation of *SchPPT_E107V_* in sHJ007	This study
sHJ013	Complementation of *SchPPT_E107M_* in sHJ007	This study
DH5α/BT340	*E. coli* strain used for excising the DNA between two FRT site	
BW25113/pIJ790	*E. coli* strain used for PCR-targeted mutagenesis	[26]
ET12567/pUZ8002	Methylation-deficient *E. coli* for conjugation	[43]
plasmids/cosmids		
pHJ0021	*scn ACP0-2* cloned as a *Nde*I-*Hin*dIII fragment into pET28a	[4]
pYY0040	Deletion of both His-Tag gene and Nus-Tag gene from pET44a	This study
pYY0041	*SchPPT* cloned as a *Nde*I-*Hin*dIII fragment into pET44a	This study
pYY0042	*SchPPT_D105A_* cloned as a *Nde*I-*Hin*dIII fragment into pET44a	This study
pYY0043	*SchPPT_E151A_* cloned as a *Nde*I-*Hin*dIII fragment into pET44a	This study
pYY0044	*SchPPT_E107A_* cloned as a *Nde*I-*Hin*dIII fragment into pET44a	This study
pYY0045	*SchPPT_E107V_* cloned as a *Nde*I-*Hin*dIII fragment into pET44a	This study
pYY0046	*SchPPT_E107M_* cloned as a *Nde*I-*Hin*dIII fragment into pET44a	This study
pHJ0024	*SchPPT* cloned as a *Nde*I-*Hin*dIII fragment into pET28a	[4]
pYY0062	*SchPPT_D105A_* cloned as a *Nde*I-*Hin*dIII fragment into pET28a	This study
pYY0063	*SchPPT_E151A_* cloned as a *Nde*I-*Hin*dIII fragment into pET28a	This study
pHJ0030	Derived from 35E12, *SchPPT* was replaced with *aac(3)IV*	[4]
pHJ0034	Derived from pHJ0030, *SchPPT* was in-frame deleted	This study
pIJ8660	A site-specific integration vector containing ermEp*, Ф31 *int* and *attP*	[27]
pHJ0033	Derived from pIJ8660 with *SchPPT* under the control of ermEp*	[4]
pYY0047	*SchPPT_D105A_* cloned as a *Nde*I-*Hin*dIII fragment into pIJ8660	This study
pYY0048	*SchPPT_E151A_* cloned as a *Nde*I-*Hin*dIII fragment into pIJ8660	This study
pYY0049	*SchPPT_E107A_* cloned as a *Nde*I-*Hin*dIII fragment into pIJ8660	This study
pYY0050	*SchPPT_E107V_* cloned as a *Nde*I-*Hin*dIII fragment into pIJ8660	This study
pYY0051	*SchPPT_E107M_* cloned as a *Nde*I-*Hin*dIII fragment into pIJ8660	This study
pHJ0029	*sch FAS ACP* cloned as *Nde*I-*Hin*dIII fragment into pET28a	[4]
pYY0052	*Hppt* cloned as a *Nde*I-*Hin*dIII fragment into pET44a	This study
pYY0064	*Hppt_D112A_* cloned as a *Nde*I-*Hin*dIII fragment into pET44a	This study
pYY0065	*Hppt_E114A_* cloned as a *Nde*I-*Hin*dIII fragment into pET44a	This study
pYY0066	*Hppt_E155A_* cloned as a *Nde*I-*Hin*dIII fragment into pET44a	This study
pYY0060	*Sppt* cloned as a *Nde*I-*Hin*dIII fragment into pET28a	This study
pYY0061	*Sppt_Q112E_* cloned as a *Nde*I-*Hin*dIII fragment into pET28a	This study

*SchPPT* was digested with *Nde*I/*Hin*dIII from plasmid pHJ0024 [4], in which *SchPPT* was cloned as a *Nde*I/*Hin*dIII fragment into pET28a (Novagen), into the same sites of pET44a, yielding plasmid pYY0041. Each gene of five point mutants of SchPPT was amplified by mutagenesis PCR (QuikChange Site-Directed Mutagenesis Kit, Stratagene) from pYY0041 as template and primers HJ0117-HJ0122 and HJ0147-HJ0150, respectively. Each of the five genes was cloned into pET44a, yielding plasmids pYY0042-pYY0046, respectively. Co-expression of each plasmid pYY0041-pYY0046 with pHJ0021 in *E. coli*, and purification and LC-MS analysis of *scn* ACP0-2 are performed according to the procedures described above.

### In vitro phosphopantetheinylation of *scn* ACP0-2 catalyzed by SchPPT, SchPPT_D105A,_ or SchPPT_E151A_

*SchPPT_D105A_* and *SchPPT_E151A_* were cloned as *Nde*I/*Hin*dIII fragments from pET44a into pET28a, yielding plasmids pYY0062 and pYY0063, respectively. Each of the two plasmids was introduced into *E. coli* BL21(DE3) to overproduce proteins as N-terminally His_6_-tagged proteins under the induction with 0.4 mM IPTG at 30 °C for 4 h. A typical phosphopantetheinylation reaction mixture of 0.1 ml containing 100 mM Tris-HCl (pH 7.5), 1.25 mM MgCl_2_, 2.5 mM tris(carboxyethyl)phosphine hydrochloride (TCEP), 200 µM ACP, 20 µM PPTase, and 2 mM CoA was incubated at 25°C for 30 min. The reactions were quenched by freezing reaction mixtures with dry ice. LC-MS analysis of ACP was performed as described previously [4].

### In vivo gene complement system

The *SchPPT* in-frame deletion mutant was constructed by using PCR targeting system as follows [26]. The cosmid pHJ0030 [4], in which *SchPPT* was replaced with *aac(3)IV*, was transferred into *E. coli* DH5α/BT340 to excise the *aac(3)IV* gene, resulting in cosmid pHJ0034. After conjugal transfer of pHJ0034 from *E. coli* ET12567/pUZ8002 into *S. chattanoogensis* L10, exconjugants were obtained after selection for thiostrepton. Exconjugants were then inoculated onto YMG plates for two rounds of nonselective growth before selection by replica plating for thiostrepton-sensitive colonies. The resulting strain, in which *SchPPT* was in-frame deleted, was designated as sHJ007 and confirmed by PCR analysis using primers HJ0077/HJ0078.

A site-specific integration vector pIJ8660 [27], containing ermEp* promoter, Ф31 *int*, and *attP*, was used to construct an integration recombinant plasmid. The *Nde*I/*Not*I DNA fragments of *SchPPT_D105A_*, *SchPPT_E107A_*, *SchPPT_E107V_*, *SchPPT_E107M_*, and *SchPPT_E151A_* were cloned from pYY0042-pYY0046 into the same sites of pIJ8660, resulting in the plasmids pYY0047-pYY0051. The pYY0047-pYY0051 and pHJ0033 [4] were transferred into sHJ007 via conjugal transfer from *E. coli* ET12567/pUZ8002 using standard procedures. The resulting strains were designated as sHJ008-sHJ013 and confirmed by PCR analyses using primers Pri53/WYY0014. Fermentation of *S. chattanoogensis* L10 and its recombinant strains and quantification of natamycin production were performed in triplicate as described previously [4], [28]–[30].

### Co-expression of *sch FAS ACP* with *Hppt* or each of Hppt point mutant genes

The plasmid pYY0040 was introduced into *E. coli* BL21(DE3) containing pHJ0029 [4], in which *sch FAS ACP* was cloned as a *Nde*I/*Hin*dIII fragment into pET28a. BL21(DE3)/pHJ0029/pYY0040 was induced with 0.4 mM IPTG at 30 °C for 4 h to overproduce *sch* FAS ACP. S*ch* FAS ACP was purified by affinity chromatography on Ni-NTA agarose and then dialyzed against 20 mM Tris·HCl (pH8.0), 25 mM NaCl, 1 mM dithiothreitol (DTT), and 10% glycerol. S*ch* FAS ACP was analyzed by HPLC as described previously [4].

Protein sequence of Hppt was obtained from NCBI database. Codons of the encoding gene were changed into the preferred codons of *E. coli.* The correspondence DNA sequence was chemically synthesized and cloned into the *Nde*I/*Hin*dIII sites of pET44a, yielding plasmid pYY0052. Each of three Hppt point mutant genes was amplified by mutagenesis PCR from pYY0052 as template and primers H112-H115(F/R), yielding plasmids pYY0064-pYY0066, respectively. Co-expression of each plasmid (pYY0052, pYY0064-pYY0066) with pHJ0029 in *E. coli*, purification and HPLC analysis of *sch* FAS ACP were performed according to the procedures described above.

### In vitro phosphopantetheinylation of *sch* FAS ACP catalyzed by Sppt or Sppt_Q112E_

Protein sequence of Sppt was obtained from NCBI database. Codons of the encoding gene were changed into the preferred codons of *E. coli.* The correspondence DNA sequence was chemically synthesized and cloned into the *Nde*I/*Hin*dIII sites of pET28a, yielding plasmid pYY0060. The *Sppt_Q112E_* gene was amplified by mutagenesis PCR from pYY0060 as template and primers FK161/FK162 and cloned into the *Nde*I/*Hin*dIII sites of pET28a, yielding plasmid pYY0061. BL21(DE3)/pYY0060 and BL21(DE3)/pYY0061 were induced with 0.4 mM IPTG at 37°C for 4 h to overproduce Sppt and Sppt_Q112E_, respectively. The proteins were purified by affinity chromatography on Ni-NTA agarose and then dialyzed against 20 mM Tris-HCl (pH 8.0), 25 mM NaCl, 1 mM DTT, and 10% glycerol.

A typical in vitro phosphopantetheinylation reaction mixture of 0.1 ml containing 100 mM Tris-HCl (pH 7.5), 1.25 mM MgCl_2_, 2.5 mM TCEP, 200 µM *sch* FAS ACP, 20 µM Sppt or Sppt_Q112E_, and 2 mM CoA was incubated at 25°C for 30 min. The reactions were quenched by freezing reaction mixtures with dry ice. HPLC analysis of *sch* FAS ACP were performed as described previously [4].

### Gene synteny analysis

We examined the chromosomal localization of PPTase homologs and neighboring genes using the Ensembl and UCSC genome browsers, with additional information obtained using the Genomicus website v70.01 (http://www.dyogen.ens.fr/genomicus-70.01?/cgi-bin/search.pl) [31] or Integrated Microbial Genomes (http://img.jgi.doe.gov/cgi-bin/w/main.cgi) [32].

### Phylogenetic analysis

Multiple sequence alignment (MSA) was carried out by using CLUSTALW and MUSCLE with the default parameter setting [33]–[34]. The alignment was then manually improved by using BioEdit 7.1.11, and the MSA generated by CLUSTALW was used as reference for manual adjustments. The best amino acid substitution model was determined with MEGA 6 to be LG+I+G+F. We constructed maximum likelihood (ML) and neighbor-joining (NJ) tree using PHYML and MEGA version 6.06 [35]–[36]. Reliability of interior branches was assessed using bootstrap support with 1000 replicates. Tree files were viewed using MEGA, and edited by Adobe Illustrator.

## Results

### Variation of the magnesium binding residues of Group II PPTases

Since the magnesium binding residues of PPTases are essential for PPTase activities, we aligned the magnesium binding residues of 556 group II PPTases from databases of GenBank, Phytozome (http://phytozome.net), and Ensembl (http://www.ensembl.org). Interestingly, group II PPTases can be classified into two subgroups based on numbers of the magnesium binding residues. The three-magnesium-binding-residue-PPTases contain three magnesium binding residues, which form the triad Asp-Glu-Glu, such as Sfp. The two-magnesium-binding-residue-PPTases contain two magnesium binding residues corresponding to the first and the third magnesium binding residues of the three-magnesium-binding-residue-PPTases, forming the triad Asp-Xxx-Glu, such as AASHDPPT. The second residues of the triad Asp-Xxx-Glu include Met, Val, Ala, Gln, Thr, Ser, Leu, and Cys. All known animal PPTases, algal PPTases, and fugal PPTases belong to two-magnesium-binding-residue-PPTases. All known animal PPTases and algal PPTases contain the triads Asp-Met-Glu and Asp-Ala-Glu, respectively. Most prokaryotic group II PPTases belong to three-magnesium-binding-residue-PPTases. All known prokaryotic two-magnesium-binding-residue-PPTases contain the triad Asp-Gln-Glu. Both three-magnesium-binding-residue-PPTases and two-magnesium-binding-residue-PPTases are found in plant. Most plant two-magnesium-binding-residue-PPTases contain the triad Asp-Val-Glu (Table 2 and Figures 1, S1, S2, S3, and S4).

**Table 2:** Distribution of group II PPTases.

	Asp-Xxx-Glu									
	Glu	Met	Val	Ala	Thr	Gln	Ser	Leu	Cys	sum
archaea	4	0	0	0	0	0	0	0	0	4
cyanobacteria	7	0	0	0	0	1	0	0	0	8
bacteria	352	0	0	0	0	9	0	0	0	361
fungi	0	12	30	12	12	0	3	1	1	71
algae	1	0	0	5	0	0	0	0	0	6
plant	20	1	18	1	0	0	0	0	0	40
animal	0	66	0	0	0	0	0	0	0	66
sum	384	79	48	18	12	10	3	1	1	556

### Effects of magnesium binding residues of a three-magnesium-binding-residue-PPTase SchPPT

Since the second magnesium binding residues of three-magnesium-binding-residue-PPTases are missing in two-magnesium-binding-residue-PPTases, we characterized effects of three magnesium binding residues of the formers to their activities. SchPPT from *S. chattanoogensis* L10 was used as a model of three-magnesium-binding-residue-PPTases. SchPPT is necessary to natamycin biosynthesis since it catalyzes the phosphopantetheinylation of *scn* ACPs (*S. chattanoogensis* natamycin biosynthetic acyl carrier proteins) in natamycin biosynthetic PKS [4]. We constructed five point mutants of SchPPT. The first magnesium binding residue D105 and the third magnesium binding residue E151 in SchPPT were replaced with Ala, resulting in SchPPT_D105A_ and SchPPT_E151A_, respectively. The second magnesium binding residue E107 was replaced with Ala, Val, and Met, resulting in SchPPT_E107A_, SchPPT_E107V_, and SchPPT_E107M_, respectively (Figure 1).

An in vitro co-expression system was built up to characterize activities of point mutants of SchPPT. *Scn* ACP0-2, the second ACP domain in the loading module of *scn* PKS, was used as a substrate of SchPPT and the point mutants [4]. *Scn ACP0-2* in pET28a was co-expressed with pYY0040, in which both His-tag gene and Nus-Tag gene were deleted from pET44a, in *E. coli*. LC-MS data showed *scn* ACP0-2 produced from *E. coli* contained only apo-proteins, which was consistent with the results reported previously [4]. Then *scn ACP0-2* in pET28a was co-expressed with *SchPPT* in pET44a in *E. coli*. LC-MS data showed *scn* ACP0-2 contained both apo-proteins and holo-proteins, indicating SchPPT could phosphopantetheinylate *scn* ACP0-2 under these conditions. Finally *scn ACP0-2* in pET28a was co-expressed with each of the SchPPT point mutant genes in pET44a in *E. coli*. LC-MS data showed both SchPPT_D105A_ and SchPPT_E151A_ lost their activities to phosphopantetheinylate *scn* ACP0-2. However, SchPPT_E107A_, SchPPT_E107V_, and SchPPT_E107M_ were still active (Figure S5).

To exclude the possibility that abolishment of activities of SchPPT_D105A_ and SchPPT_E151A_ due to mis-folding of proteins or no expression of genes, both mutants were produced in *E. coli* as His-tagged proteins and purified to homogeneities. In vitro phosphopantetheinylation of *scn* ACP0-2 was performed by incubation of *scn* ACP0-2 with CoA and each of the mutants by using wild type SchPPT as a positive control as described previously [4]. LC-MS data showed only wild type SchPPT but neither of the two mutants phosphopantetheinylated *scn* ACP0-2 under these conditions (Figure S6).

**Figure 2:**
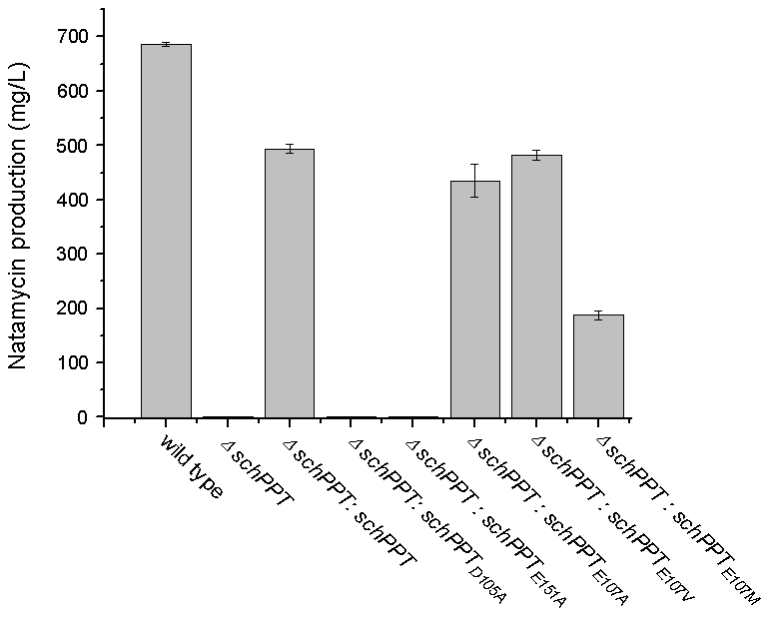
Natamycin production of *S. chattanoogensis* L10 and its recombinant strains. Wild type (*S. chattanoogensis* L10), *ΔschPPT* (sHJ007), *ΔschPPT:schPPT* (sHJ008), *ΔschPPT:schPPT_D105A_* (sHJ009), *ΔschPPT:schPPT_E151A_* (sHJ010), *ΔschPPT:schPPT_E107A_* (sHJ011), *ΔschPPT:schPPT_E107V_* (sHJ012), *ΔschPPT:schPPT_E107M_* (sHJ013).

An in vivo gene complement system was also built up to characterize the activity of SchPPT point mutants. We in-frame deleted *SchPPT* in *S. chattanoogensis* L10, resulting in strain sHJ007. Fermentation of sHJ007 in YEME liquid medium in triplicate showed sHJ007 lost ability to produce natamycin, confirming the activity of SchPPT is essential to natamycin production. Then we complemented *SchPPT* under the control of the ermEp* promoter in the sHJ007, resulting in strain sHJ008. Fermentation of sHJ008 in YEME liquid medium in triplicate showed sHJ008 produced natamycin with the yield of 494 mg/L at 96 h, indicating SchPPT could complement sHJ007 under these conditions. Finally we complemented each of five SchPPT point mutant genes under the control of the ermEp* promoter in the sHJ007, resulting in strain sHJ009-sHJ0013. Fermentation data showed complementation of neither SchPPT_D105A_ nor SchPPT_E151A_ could produce natamycin. However, complementation of SchPPT_E107A_, SchPPT_E107V_, and SchPPT_E107M_ produced natamycin at 96 h with the yield of 434 mg/L, 482 mg/L, and 188 mg/L, respectively (Figure 2). Both in vitro and in vivo data herein reveal that the first and the third magnesium binding residues in SchPPT are essential for enzyme activity; however, the second magnesium binding residues is non-essential.

### Effects of magnesium binding residues of a three-magnesium-binding-residue-PPTase Hppt

**Figure 3:**
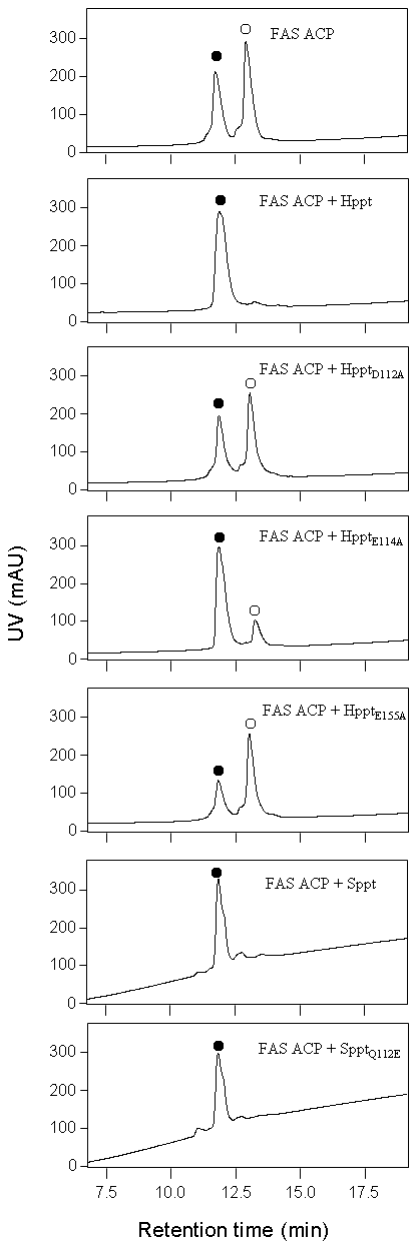
In vitro phosphopantetheinylation of *sch* FAS ACP catalyzed by Hppt, Sppt, and point mutants. ○, apo-form; •, holo-form; mAU, milli-absorbance units.

Hppt, a single PPTase in *Haemophilus influenza*, was also used as a model of three-magnesium-binding-residue-PPTases. *Hppt*, in which all codons of wild type gene were changed into the preferred codons of *E. coli*, was chemically synthesized and cloned into pET44a, yielding plasmid pYY0052. The ACP of FAS in *S. chattanoogensis* L10, *sch* FAS ACP, was used as the substrate of Hppt. Plasmid pHJ0029 [4], in which *sch FAS ACP* was cloned into pET28a, was co-expressed with pYY0040 in *E. coli*. HPLC data showed *sch* FAS ACP produced from *E. coli* contained both apo-proteins and holo-proteins (Figure 3), which was consistent with the results that *E. coli* ACPS could phosphopantetheinylate *sch* FAS ACP incompletely [4]. *Sch FAS ACP* in pET28a was then co-expressed with pYY0052 in *E. coli*. HPLC data showed *sch* FAS ACP contained only holo-proteins, indicating Hppt could phosphopantetheinylate *sch* FAS ACP under these conditions. We finally constructed three point mutants of Hppt. The magnesium binding residues (D112, E114, and E155) were replaced with Ala, resulting in Hppt_D112A_, Hppt_E114A_, and Hppt_E155A_, respectively (Figure S1). *Sch FAS ACP* in pET28a was then co-expressed with each of the Hppt point mutant genes in pET44a in *E. coli*. Among of three Hppt point mutants, only Hppt_E114A_ remained this activity (Figure 3).

### Construction of a three-magnesium-binding-residue-PPTase mimic based on a two-magnesium-binding-residue-PPTase Sppt

Sppt, a single PPTase in *Synechocystis* sp. PCC6803, was used as a model of two-magnesium-binding-residue-PPTases. It has been reported that Sppt phosphopantetheinylates ACPs of type II FASs but not ACPs from secondary metabolism [20]. S*ppt*, in which all codons of wild type gene were changed into the preferred codons of *E. coli*, was chemically synthesized and cloned into pET28a. Sppt was produced in *E. coli* and then purified to homogeneity. *Sch* FAS ACP was also used as the substrate of Sppt. After incubation of *sch* FAS ACP with CoA in the presence of Sppt, HPLC analysis showed all apo-proteins converted into holo-proteins, indicating Sppt could phosphopantetheinylate *sch* FAS ACP. We constructed a three-magnesium-binding-residue-PPTase mimic based on Sppt. The second residue in the triad of Sppt, Q112, was replaced with Glu, resulting in a three-magnesium-binding-residue-PPTase mimic Sppt_Q112E_ (Figure 1). After incubation of *sch* FAS ACP with CoA in the presence of Sppt_Q112E_, HPLC analysis showed all apo-proteins converted into holo-proteins, indicating mutation of Sppt into a three-magnesium-binding-residue-PPTase mimic remained its activity (Figure 3).

### Gene synteny and gene duplication

**Figure 4:**
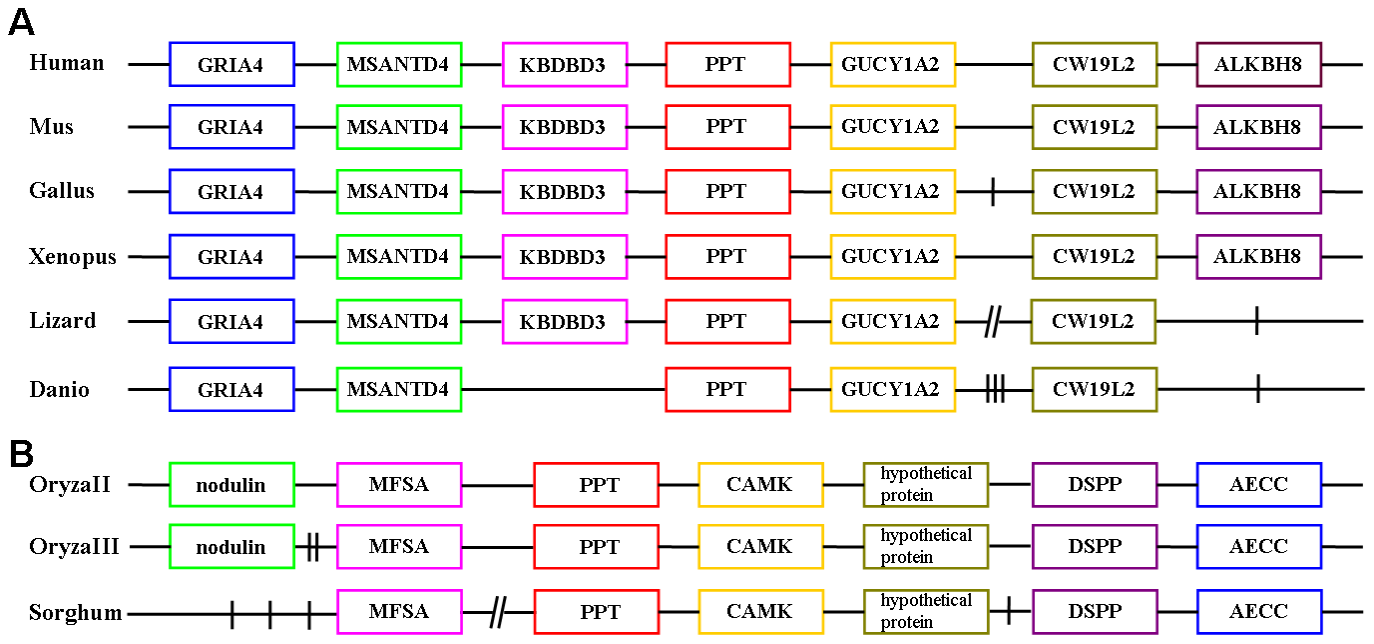
Gene synteny of PPTases in vertebrates (A) and some plants (B). GRIA4: glutamate receptor, ionotropic, AMPA 4; MSANTD4: Myb/SANT-like DNA-binding domain containing 4 with coiled-coils; KBDBD3: kelch repeat and BTB (POZ) domain containing 3; PPT: phosphopantetheinyl transferase; GUCYIA2: guanylate cyclase 1, soluble, alpha 2; CW19L2: CWF19-like 2, cell cycle control; ALKBH8: alkB, alkylation repair homolog 8; CU694319.1: Uncharacterized protein; MFSA: major facilitator superfamily antiporter; CAMK: calcium/calmodulin depedent protein kinases; DSPP: dual specificity protein phosphatase; AECC: auxin efflux carrier component. One vertical line, two vertical lines, and three vertical lines represented one gene, two genes, and three genes, respectively. The diagonal lines represented more than three genes.

To study colinearity of group II PPTases, 56 PPTases from representative species were selected for gene synteny analysis, including 4 archaeal PPTases, 4 cyanobacterial PPTases, 14 bacterial PPTases, 11 fungal PPTases, 3 algal PPTases, 10 plant PPTases, and 10 animal PPTases (Table 3 and Figures S7, S8, S9, and S10). Except six animal PPTases and three plant two-magnesium-binding-residue-PPTases, the other PPTases don’t show any gene synteny conservation (Figure 4).

**Table 3:** The 56 selected PPTases for phylogenetic analysis.

	Protein	Organism	Accession number	Ref.
archaea	Methanomethylovorans	*Methanomethylovorans hollandica* DSM 15978	YP_007312575.1	
	Methanobrevibacter	*Methanobrevibacter ruminantium* M1	YP_003423257.1	[1]
	Thaumarchaeota	*Thaumarchaeota archaeon* SCGC AB-539-E09	ZP_23951850.1	
	Methanocella	*Methanocella paludicola* SANAE	YP_003355289.1	[1]
cyano- bacterium	Sppt	*Synechocystis* sp. PCC 6803	BAA10326	[20]
	Pleurocapsa	*Pleurocapsa* sp. PCC 7327	YP_007079598.1	
	Oscillatoria	*Oscillatoria acuminata* PCC 6304	YP_007086947.1	
	PPT_Ns_	*Nodularia spumigena*	AAY42632.1	[21]
bacterium	SchPPT	*Streptomyces chattanoogensis* L10	AFF18625.1	[4]
	Sfp	*Bacillus subtilis* subsp. Subtilis str. 168	CAA44858.1	[23]–[24]
	Myxococcus	*Myxococcus fulvus* HW-1	YP_004668005.1	
	Pelosinus	*Pelosinus fermentans* DSM 17108	ZP_10325243.1	
	SCO5883 (RedU)	*Streptomyces coelicolor* A3(2)	NP_630004.1	[6]
	SCO6673	*Streptomyces coelicolor* A3(2)	NP_630748.1	[6]
	Gluconacetobacter	*Gluconacetobacter diazotrophicus* PAl 5	YP_001603066.1	
	Granulicella	*Granulicella mallensis* MP5ACTX8	YP_005057339.1	
	Rhodopirellula	*Rhodopirellula sallentina* SM41	ZP_23722709.1	
	Odoribacter	*Odoribacter splanchnicus* DSM 20712	YP_004254021.1	
	EntD	*Escherichia coli* str. K-12 substr. MG1655	NP_415115.2	[7]
	Sorangium	*Sorangium cellulosum* So ce56	YP_001615530.1	
	Clostridium	*Clostridium acetobutylicum* ATCC 824	NP_347957.1	
	OvmF	*Streptomyces antibioticus*	CAG14972.1	[44]
fungus	Schizosaccharomyces	*Schizosaccharomyces pombe* 972h	NP_594603.1	
	Sordaria	*Sordaria macrospora* k-hell	CCC07706.1	
	Baudoinia	*Baudoinia compniacensis* UAMH 10762	EMC94199.1	
	Punctularia	*Punctularia strigosozonata* HHB-11173 SS5	EIN10680.1	
	Cryptococcus	*Cryptococcus neoformans* var. grubii H99	AFR96401.1	
	Mixia	*Mixia osmundae* IAM 14324	GAA96409.1	
	Wallemia	*Wallemia sebi* CBS 633.66	EIM24045.1	
	Emericella	*Emericella nidulans*	AAF12814.1	
	LYS5	*Saccharomyces cerevisiae*	CAA96866.1	[40]
	Claviceps	*Claviceps purpurea* 20.1	CCE33539.1	
	Tetrapisispora	*Tetrapisispora phaffii* CBS 4417	CCE62507.1	
algae	Coccomyxa	*Coccomyxa subellipsoidea* C-169	EIE27690.1	
	Micromonas	*Micromonas* sp. RCC299	XP_002501796.1	
	Ostreococcus	*Ostreococcus tauri*	XP_003080746.1	
plant	Populus	*Populus trichocarpa*	Potri.016G078400.1	
	Arabidopsis	*Arabidopsis thaliana*	NP_974284.2	
	OryzaI	*Oryza sativa Japonica Group*	NP_001061345.1	
	Selaginella	*Selaginella moellendorffii*	XP_002969109.1	
	Vitis	*Vitis vinifera* (wine grape)	XP_002274180.2	
	Solanum	*Solanum lycopersicum*	XP_004246501.1	
	Glycine	*Glycine max*	XP_003518982.1	
	Sorghum	*Sorghum bicolor*	XP_002448942.1	
	OryzaII	*Oryza sativa Japonica Group*	NP_001065690.1	
	OryzaIII	*Oryza sativa Japonica Group*	NP_001066088.1	
animal	CaenorhabditisX	*Caenorhabditis elegans*	T04G9.4.1	
	CaenorhabditisV	*Caenorhabditis elegans*	T28H10.1.1	
	Drosophila	*Drosophila melanogaster*	NP_729788.1	
	Branchiostoma	*Branchiostoma floridae*	XP_002611588.1	
	Danio	*Danio rerio*	NP_001028901.1	
	Xenopus	*Xenopus* (Silurana) *tropicalis*	NP_001120584.1	
	Gallus	*Gallus gallus* (chicken)	XP_417169.2	
	Lizard	*Anolis carolinensis*	ENSACAG00000011121	
	Mus	*Mus musculus* (house mouse)	AAH30043.1	
	AASHDPPT	*Homo sapiens* (human)	Q9NRN7.2	[25]

Some organisms contain more than one copy of group II PPTase encoding genes. The genome of *Oryza sativa Japonica Group* contains three group II PPTase encoding genes, *oryzaI*, *oryzaII*, and *oryzaIII* within the chromosome 8, the chromosome 11, and the chromosome 12, respectively. OryzaII and OryzaIII may derive from gene duplication, since both of them have the same triad Asp-Val-Glu, high DNA/protein sequence similarity/identity, and conserved gene synteny at their encoding loci. However, OryzaI may come from a different origin with OryzaII and OryzaIII, since OryzaI has a different triad Asp-Glu-Glu, low DNA/protein similarity/identity comparing with OryzaII/QryzaIII, and has no gene synteny with OryzaII/QryzaIII.

The genome of *Streptomyces coelicolor* A3(2) contains two group II PPTase encoding genes, *sco5883*/*sco6673*. It is known that: SCO5883 (also known as RedU) phosphopantetheinylates RedO (ACP involved in prodiginines biosynthesis); SCO6673 phosphopantetheinylates PCPs involved in the lipopeptide calcium-dependent antibiotic (CDA) biosynthesis; and SCO4744 (group I PPTase) phosphopantetheinylates both ACP of FAS and *act* ACP (ACP involved in actinorhodin biosynthesis) [6], [37]. The genome of *E. coli* K12 contains two group II PPTases encoding genes, *entD/acpT*. It has been reported that: EntD phosphopantetheinylates EntF (PCP involved in enterobactin biosynthesis); AcpT may phosphopantetheinylate the ACPs of a FAS-like synthase; and AcpS (group I PPTase) phosphopantetheinylates ACP of FAS [1], [7], [38]. The genome of *Caenorhabditis elegans* contains two group II PPTases encoding genes, *celegansV/celegansX*. The DNA/protein sequence similarity/identity of SCO5883/SCO6673, EntD/AcpT, and CelegansV/CelegansX are low, respectively, suggesting they may not derived from gene duplication (Table 4).

**Table 4:** The DNA/protein sequence similarity/identity of PPTases.

	Protein sequence similarity/identity	DNA sequence identity (including introns)
OryzaII/OryzaIII	96%/94%	92%
OryzaI/OryzaII	43%/24%	No identity
OryzaI/OryzaIII	42%/24%	No identity
SCO5883/SCO6673	43%/21%	No identity
EntD/AcpT	43%/28%	No identity
CelegansV/CelegansX	52%/36%	No identity

### Phylogenetic relationships between the PPTases

**Figure 5:**
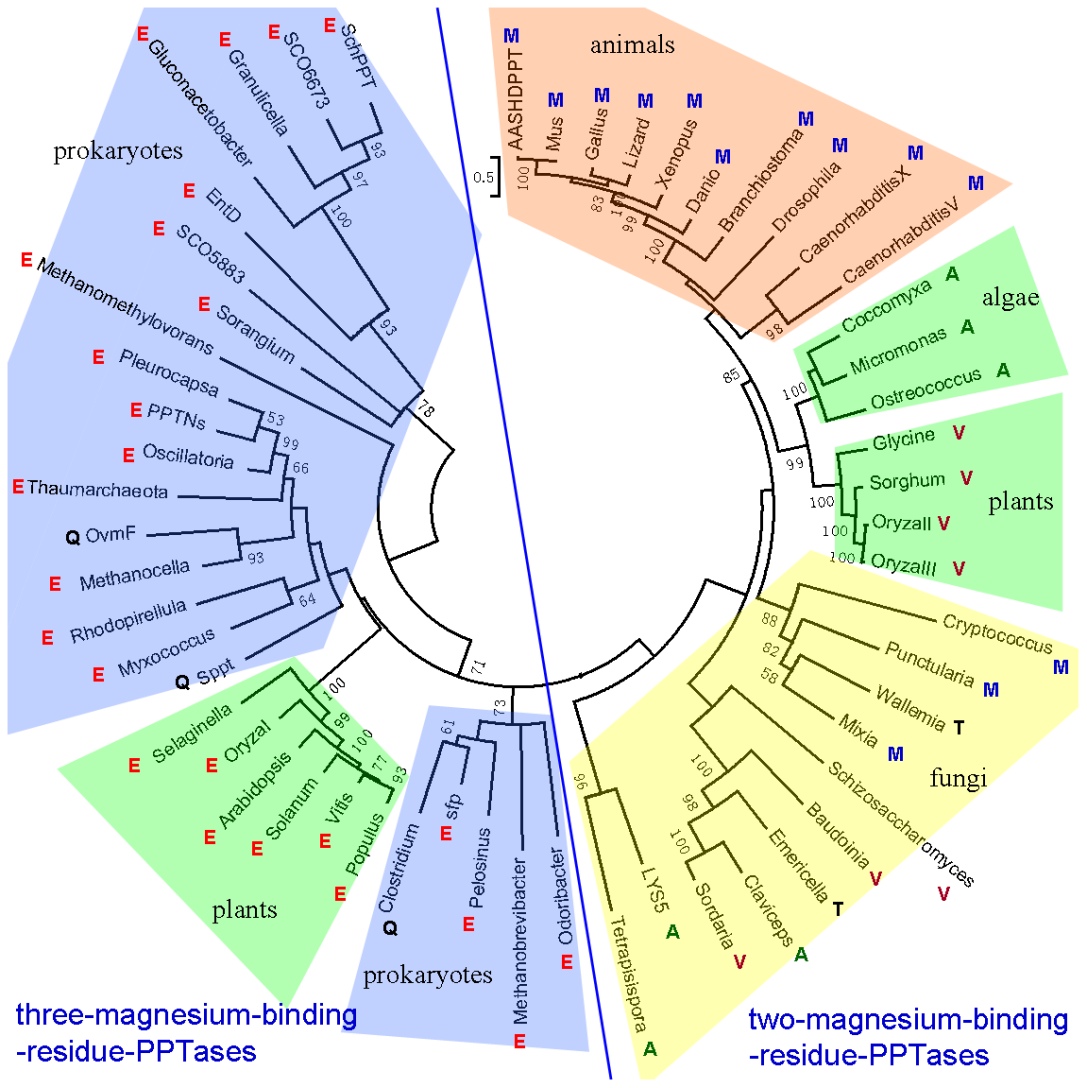
The mid-point unrooted phylogenetic tree of the 56 selected PPTases. Numbers at the branches indicate bootstrap and posterior probability values. Each colored single-letter amino acid next to the name of PPTases represents the second residue of the triad.

To study evolutionary relationship among group II PPTases, the above selected 56 PPTases were also analyzed by different phylogenetic methods. The maximum like tree can be separated into three-magnesium-binding-residue-PPTases part and two-magnesium-binding-residue-PPTases part (Figure 5). The two-magnesium-binding-residue-PPTases part included animal PPTases, algal PPTases, fungal PPTases, and plant two-magnesium-binding-residue-PPTases. The three-magnesium-binding-residue-PPTases part included plant three-magnesium-binding-residue-PPTases and prokaryotic three-magnesium-binding-residue-PPTases. All of these 10 animal PPTases form one clade. These animal PPTases may originate from one common ancestor since (i) they have the same amino acids (Met) at the second position of the triad Asp-Xxx-Glu; (ii) they are closely related homologs in the phylogenetic tree; and (iii) the vertebrate PPTases even have gene synteny. Notably, the phylogenetic tree of the animal PPTases is consistent with animal species evolution (Figures 5 and S10). Interestingly, the 10 plant PPTases are distinctly separated into two clades, the clade of plant two-magnesium-binding-residue-PPTases and the clade of plant three-magnesium-binding-residue-PPTases. The plant two-magnesium-binding-residue-PPTases may originate from one common ancestor since (i) they have the same amino acids (Val) at the second position of the triad Asp-Xxx-Glu; (ii) they are clustered in the phylogenetic tree; (iii) and they have gene synteny. The plant three-magnesium-binding-residue-PPTases may derive from horizontal gene transfer from prokaryotes since they and most prokaryotic PPTases have the same triad Asp-Glu-Glu and are closely related homologs. Prokaryotic two-magnesium-binding-residue-PPTases are close to prokaryotic three-magnesium-binding-residue-PPTases but not to eukaryotic two-magnesium-binding-residue-PPTases.

## Discussion

To date, all known group II PPTases contain two or three magnesium binding residues. Three-magnesium-binding-residue-PPTases contain three conserved magnesium binding residues forming the triad Asp-Glu-Glu, including most prokaryotic group II PPTases and some plant group II PPTases. Two-magnesium-binding-residue-PPTases with the triad Asp-Xxx-Glu contain two conserved magnesium binding residues, which corresponding to the first and the third magnesium binding residues of the three-magnesium-binding-residue-PPTases, including most eukaryotic group II PPTases.

Here characterization of the point mutants of two three-magnesium-binding-residue-PPTases (SchPPT and Hppt) showed mutations of the first residues and the third residues in the triad abolished their activities. Our data are consistent with the results that mutations of the first residues and the third residues in the triads of Sfp, Lys5, and AASHDPPT abolished the activities or decreased the activities with more than 20-fold [23], [25], [39]–[40]. Our results here showed mutations of SchPPT and Hppt into two-magnesium-binding-residue-PPTase mimics and mutation of Sppt (a two-magnesium-binding-residue-PPTase) into a three-magnesium-binding-residue-PPTase mimic remained their activities. However, it is unknown if replacement of triad Asp-Xxx-Glu in a two-magnesium-binding-residue-PPTase with triad Asp-Glu-Glu result in a bona-fide three-magnesium-binding-residue-PPTase with lack of the structural information.

Conservations of the first and the third residues in the triads of all known PPTases and our biochemical results suggested that the first and the third residues in the triads of group II PPTases are essential to the activities. The variations of the second residues in the triads and our biochemical results suggested that the second residues in the triads are non-essential to the activities. However, although the second residues in the triads are not critical to their functions, they are conserved in animals (Met), algae (Ala), plants (Val and Glu), and most prokaryotes (Glu). Therefore, the variation in this site is not random and can be used for species classification. The fixation of the second residues in the triads in different taxa may be due to selective sweep or other evolutionary forces. Most likely, the mutations of the second Mg residue may be due to random genetic drift, and the fixation of this residue in separate clades is largely independent of fitness, which could be explained by random fixation of very slightly deleterious mutations, as suggested by neutral evolution theory. A better understand of the evolution of PPTases gene family will shed new insights into the mechanism of this important enzyme in systems level [41]–[42].

## Supporting Information

Figure S1 **Protein alignment of bacterial and cyanobacterial group II PPTases.** The red words represent the proteins selected for phylogenetic analysis or mutation analysis.
(TIF)

Figure S2 **Protein alignment of fungal group II PPTases.** The red words represent the proteins selected for phylogenetic analysis.
(TIF)

Figure S3 **Protein alignment of plant and algal group II PPTases.** The red words represent the proteins selected for phylogenetic analysis.
(TIF)

Figure S4 **Protein alignment of animal group II PPTases.** The red words represent the proteins selected for phylogenetic analysis.
(TIF)

Figure S5 **Co-expression of** ***scn ACP0-2*** **with** ***schPPT*** **and the point mutant genes of SchPPT.** (A) HPLC analyses. (B) MS analyses.
(TIF)

Figure S6 **In vitro phosphopantetheinylation of** ***scn*** **ACP0-2 catalyzed by SchPPT and the point mutants of SchPPT.** (A) HPLC analyses. (B) MS analyses.
(TIF)

Figure S7 **Cladograms of bacteria and cyanobacteria (** http://www.tolweb.org **).** The selected group II PPTases for gene synteny analysis and phylogenetic analysis are in red.
(TIF)

Figure S8 **Cladograms of fungi (** http://www.jgi.doe.gov **).** The selected group II PPTases for gene synteny analysis and phylogenetic analysis are in red.
(TIF)

Figure S9 **Cladograms of plants and algae (** http://phytozome.net **).** The selected group II PPTases for gene synteny analysis and phylogenetic analysis are in red.
(TIF)

Figure S10 **Cladograms of animals (** http://www.metazome.net **).** The selected group II PPTases for gene synteny analysis and phylogenetic analysis are in red.
(TIF)

Table S1 **Primers used in this study.**
(DOC)
