# Supplementary figures and images for: Characterization and Evolutionary Implications of the Triad Asp-Xxx-Glu in Group II Phosphopantetheinyl Transferases
Source: PLoS One. 2014 Jul 18;9(7):e103031. doi: 10.1371/journal.pone.0103031 (PMC4103896; doi:10.1371/journal.pone.0103031)

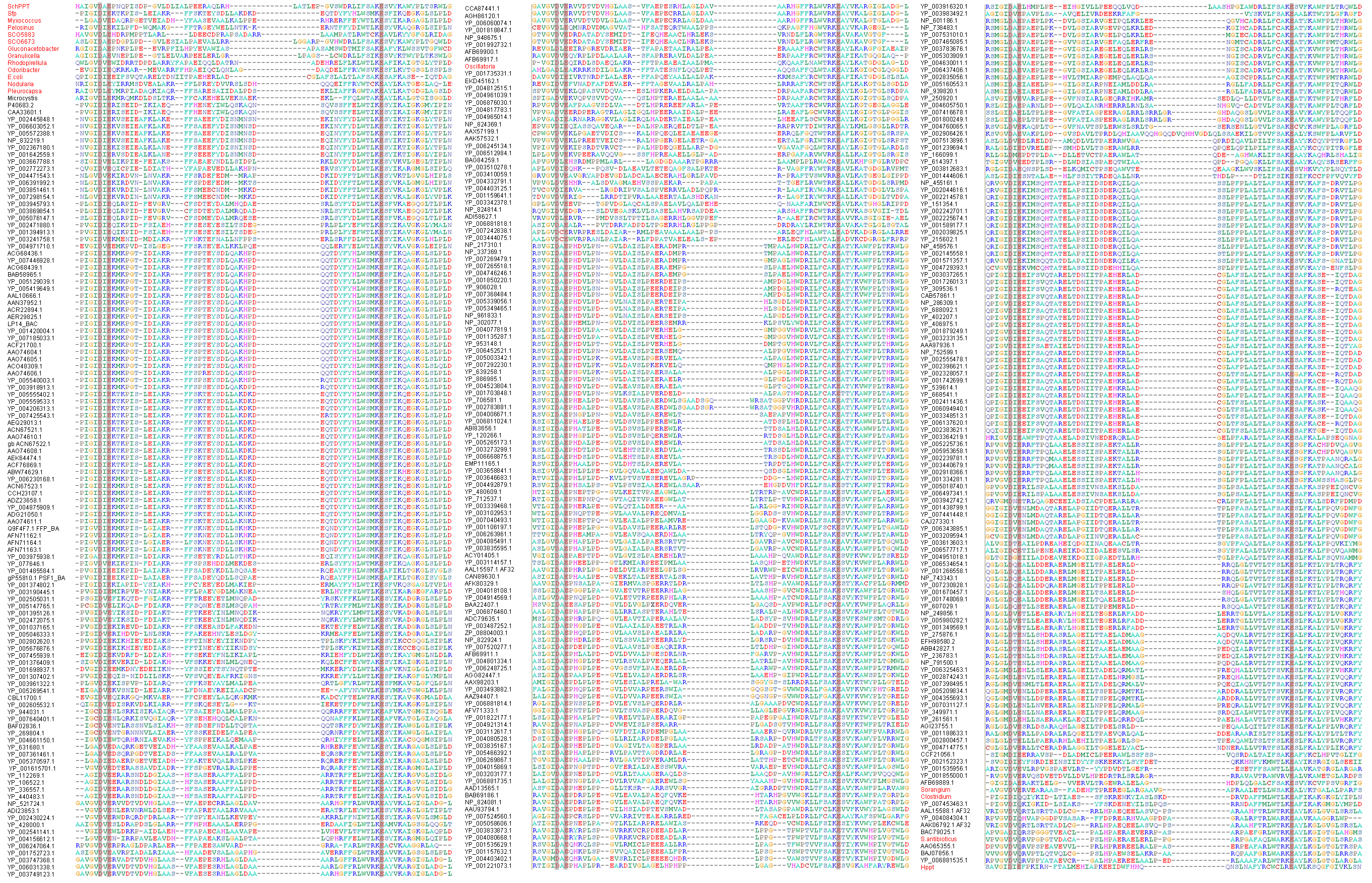

Supplement: Figure S1 — Protein alignment of bacterial and cyanobacterial group II PPTases. The red words represent the proteins selected for phylogenetic analysis or mutation analysis. (TIF) [file pone.0103031.s001.tif]

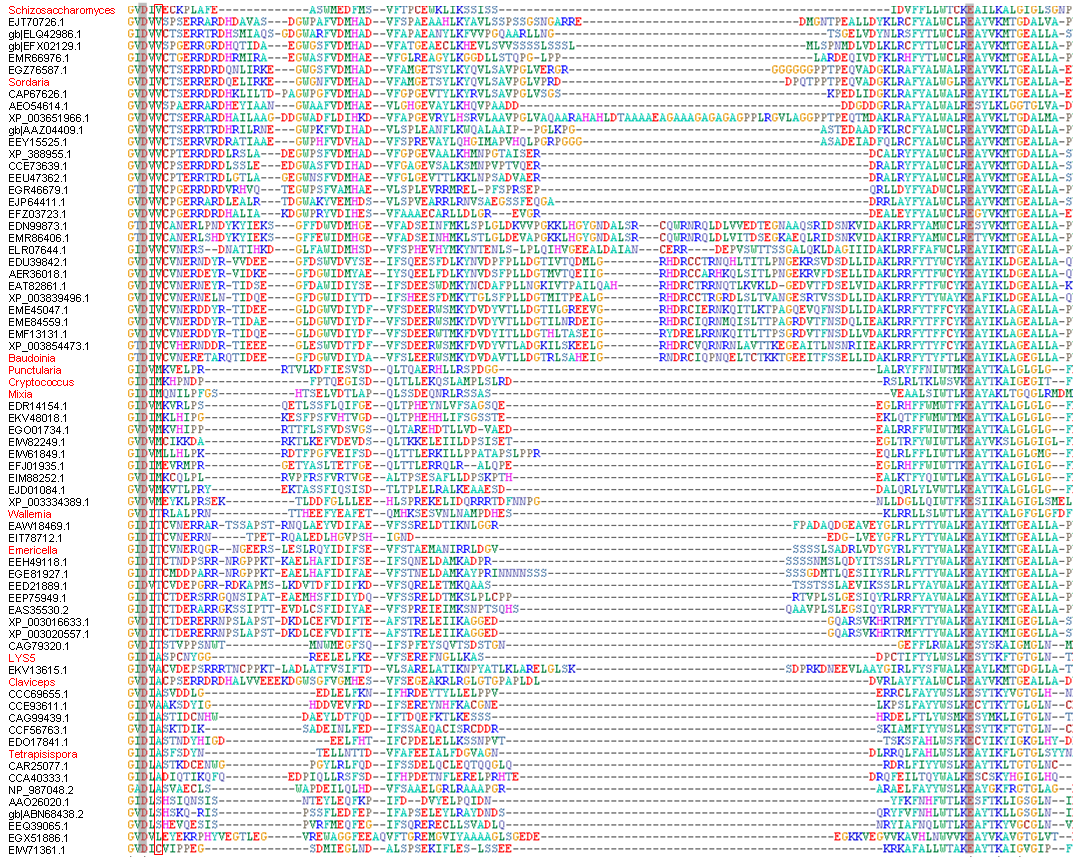

Supplement: Figure S2 — Protein alignment of fungal group II PPTases. The red words represent the proteins selected for phylogenetic analysis. (TIF) [file pone.0103031.s002.tif]

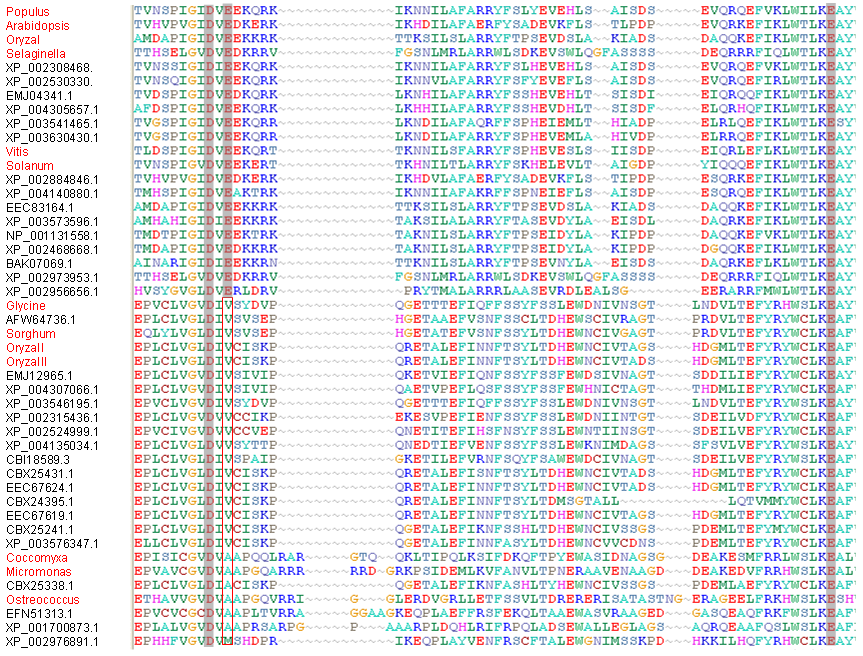

Supplement: Figure S3 — Protein alignment of plant and algal group II PPTases. The red words represent the proteins selected for phylogenetic analysis. (TIF) [file pone.0103031.s003.tif]

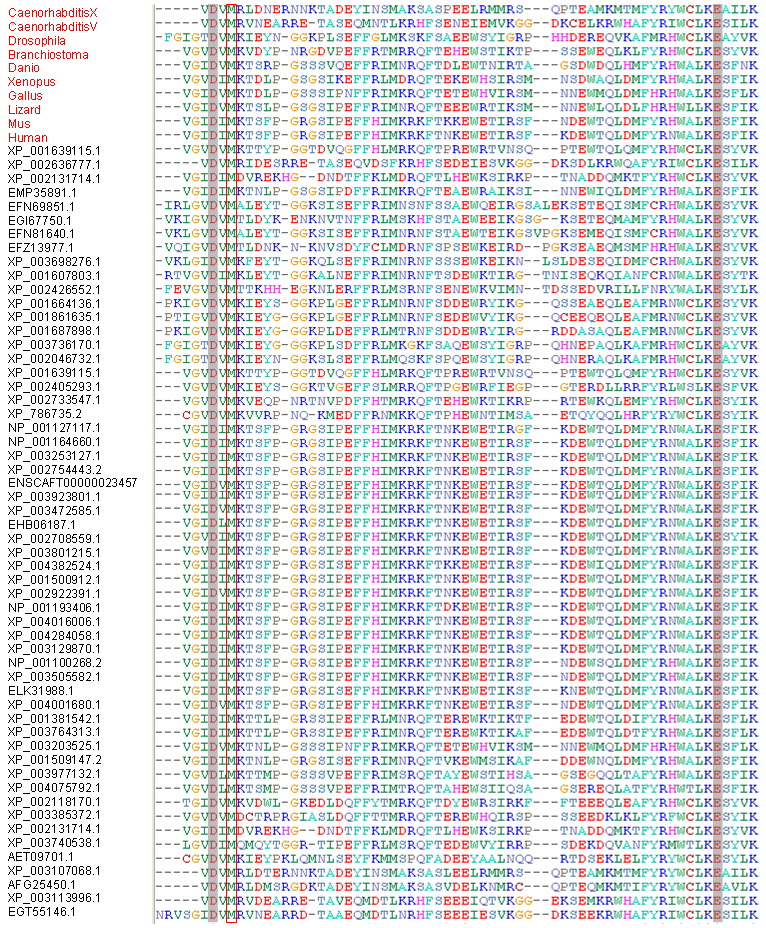

Supplement: Figure S4 — Protein alignment of animal group II PPTases. The red words represent the proteins selected for phylogenetic analysis. (TIF) [file pone.0103031.s004.tif]

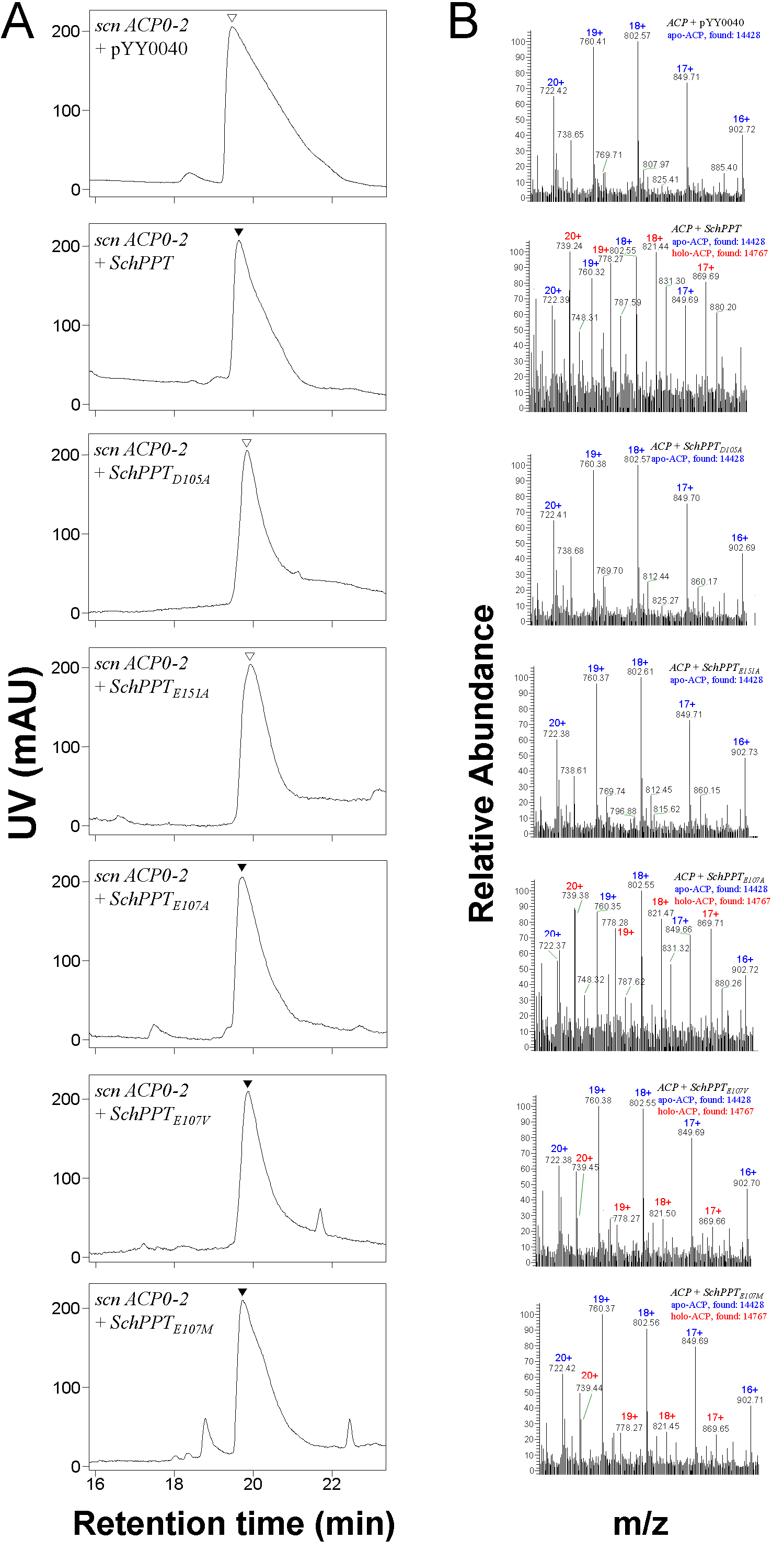

Supplement: Figure S5 — Co-expression of scn ACP0-2 with schPPT and the point mutant genes of SchPPT. (A) HPLC analyses. (B) MS analyses. (TIF) [file pone.0103031.s005.tif]

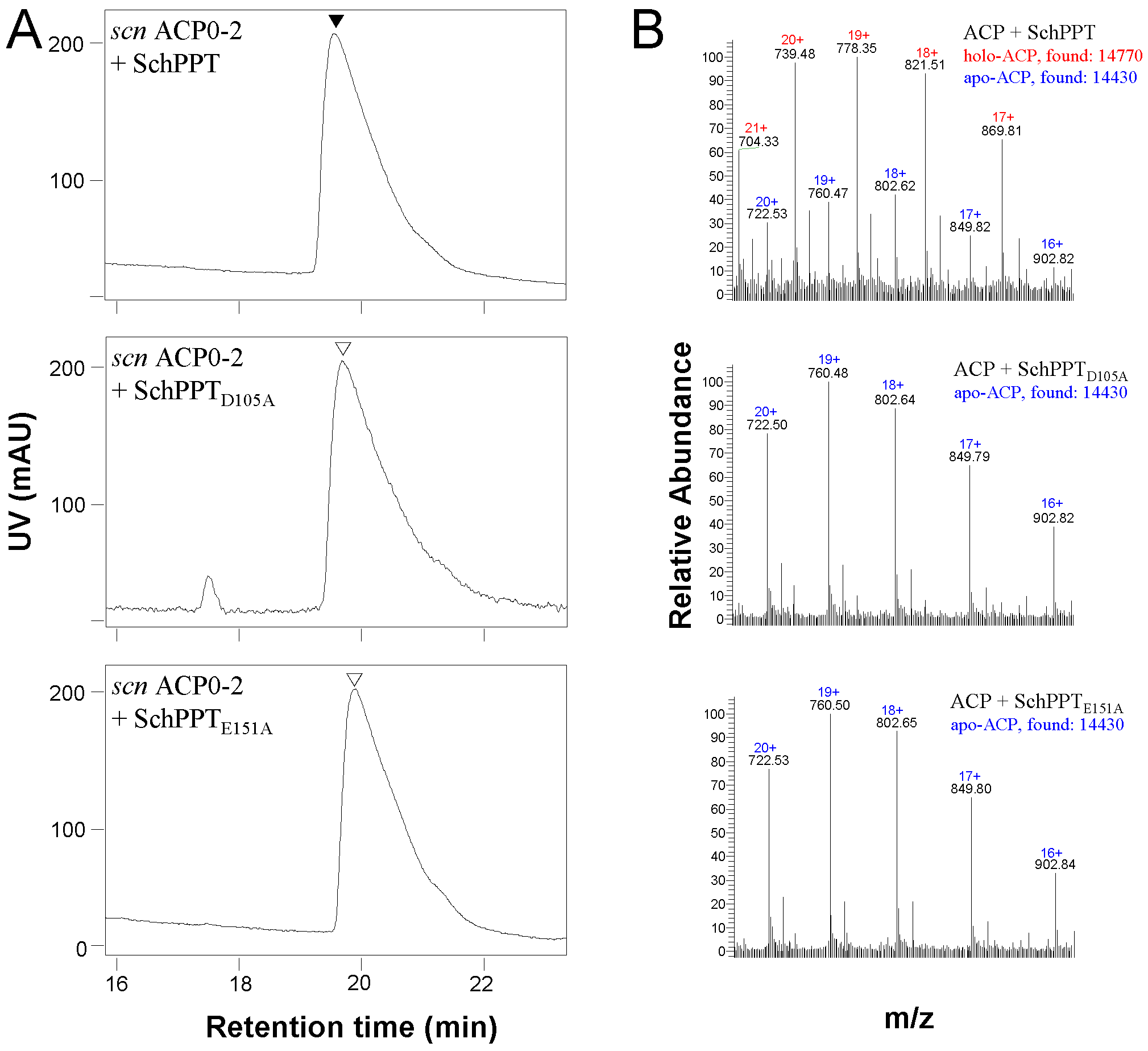

Supplement: Figure S6 — In vitro phosphopantetheinylation of scn ACP0-2 catalyzed by SchPPT and the point mutants of SchPPT. (A) HPLC analyses. (B) MS analyses. (TIF) [file pone.0103031.s006.tif]

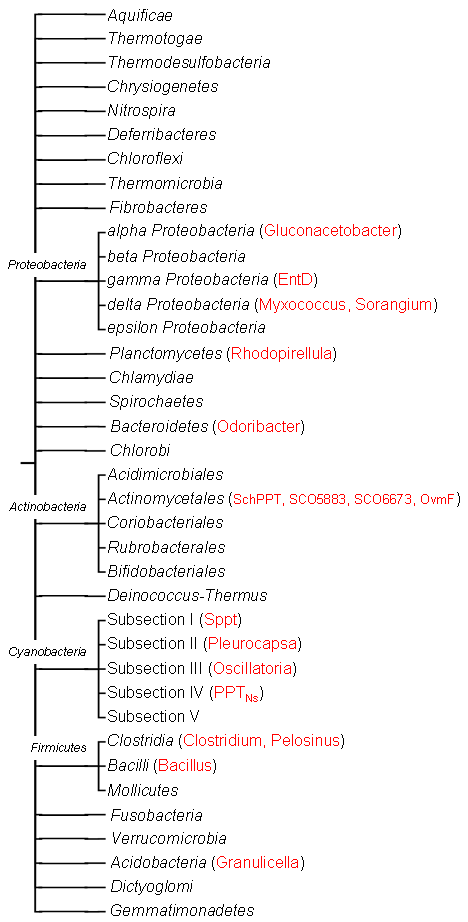

Supplement: Figure S7 — Cladograms of bacteria and cyanobacteria ( http://www.tolweb.org ). The selected group II PPTases for gene synteny analysis and phylogenetic analysis are in red. (TIF) [file pone.0103031.s007.tif]

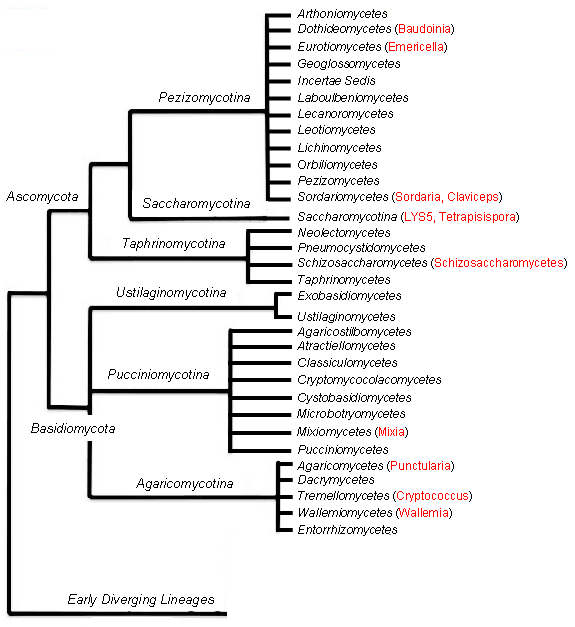

Supplement: Figure S8 — Cladograms of fungi ( http://www.jgi.doe.gov ). The selected group II PPTases for gene synteny analysis and phylogenetic analysis are in red. (TIF) [file pone.0103031.s008.tif]

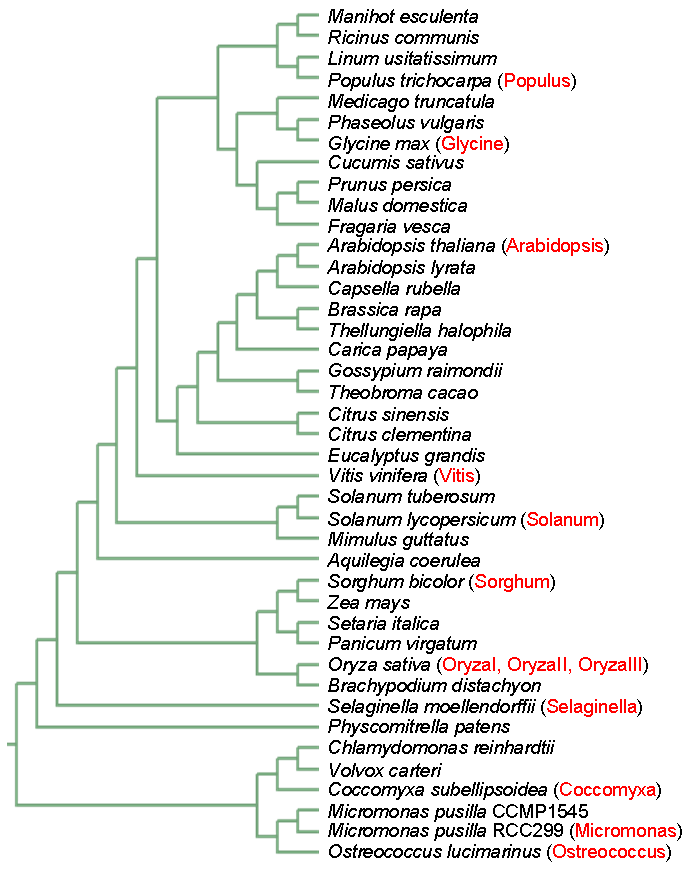

Supplement: Figure S9 — Cladograms of plants and algae ( http://phytozome.net ). The selected group II PPTases for gene synteny analysis and phylogenetic analysis are in red. (TIF) [file pone.0103031.s009.tif]

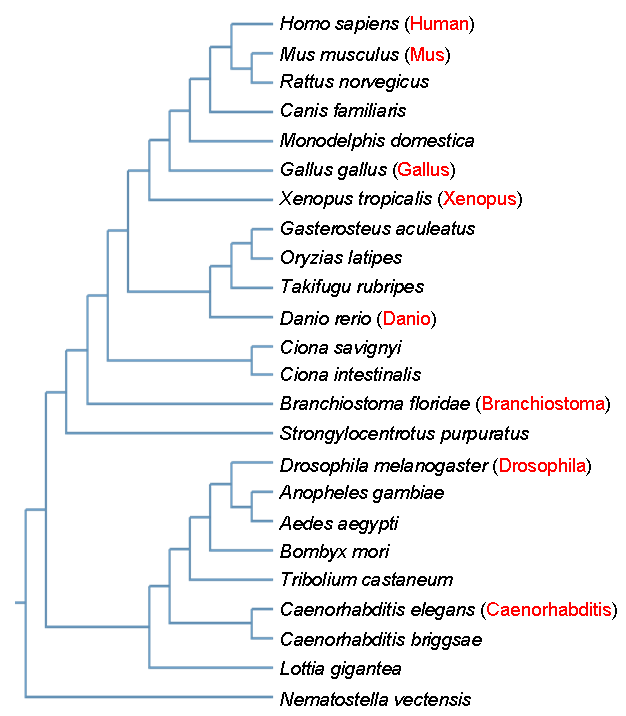

Supplement: Figure S10 — Cladograms of animals ( http://www.metazome.net ). The selected group II PPTases for gene synteny analysis and phylogenetic analysis are in red. (TIF) [file pone.0103031.s010.tif]
