# Supplementary material for: Characterization and Evolutionary Implications of the Triad Asp-Xxx-Glu in Group II Phosphopantetheinyl Transferases
Source: PLoS One. 2014 Jul 18;9(7):e103031. doi: 10.1371/journal.pone.0103031 (PMC4103896; doi:10.1371/journal.pone.0103031)
Supplement: Table S1 — Primers used in this study. (DOC) [file pone.0103031.s011.doc]

**Table S1 Primers Used in This Study**

| **Primer** | **Sequence** | **Note** |
| --- | --- | --- |
| HJ0117 | cctgcacgccatcggcgtggCcgccgaacccaaccagccca | For amplification of s*SchPPT_D105A_* |
| HJ0118 | tgggctggttgggttcggcgGccacgccgatggcgtgcagg |
|  |  |  |
| HJ0119 | cgccatcggcgtggacgccgCacccaaccagcccatcagcg | For amplification of *schPPT_E107A_* |
| HJ0120 | cgctgatgggctggttgggtGcggcgtccacgccgatggcg |
|  |  |  |
| HJ0121 | cctgatattcagcgccaaggCatccgtctacaaggcgtggt | For amplification of *schPPT_E151A_* |
| HJ0122 | accacgccttgtagacggatGccttggcgctgaatatcagg |
|  |  |  |
| HJ0147 | acgccatcggcgtggacgccATGcccaaccagcccatcagcga | For amplification of *schPPT_E107M_* |
| HJ0148 | tcgctgatgggctggttgggCATggcgtccacgccgatggcgt |
|  |  |  |
| HJ0149 | cgccatcggcgtggacgccgTacccaaccagcccatcagcg | For amplification of *schPPT_E107V_* |
| HJ0150 | cgctgatgggctggttgggtAcggcgtccacgccgatggcg |
|  |  |  |
| H112F | aaaaagcgcagtgggcattgccattgaatttccgaaaatccg | For amplification of *Hppt_D112A_* |
| H112R | cggattttcggaaattcaatggcaatgcccactgcgcttttt |
|  |  |  |
| H114F | cgcagtgggcattgatattgcctttccgaaaatccgtaactt | For amplification of *Hppt_E114A_* |
| H114R | aagttacggattttcggaaaggcaatatcaatgcccactgcg |
|  |  |  |
| H155F | tcgttgctggtgcctgcgtgccgcggttctgaaaagccaggg | For amplification of *Hppt_E155A_* |
| H155R | ccctggcttttcagaaccgcggcacgcaggcaccagcaacga |
|  |  |  |
| FK161 | gcgaaatcggcgtggacctgGaaatcatgctgccgaaaccg | For amplification of *sppt_Q112E_* |
| FK162 | cggtttcggcagcatgatttCcaggtccacgccgatttcgc |
